# Systematic Review of Interventions for LatinX and American Indian Family Dyads Coping With Chronic Illness

**DOI:** 10.1093/geroni/igaa057.325

**Published:** 2020-12-16

**Authors:** Michael McCarthy, Angelica Sanchez, Yolanda Evie Garcia, Dorothy Dunn, Heather Williamson, Julie Baldwin, Morgan Lee-Regalado Hustead, Tamilyn Bakas

**Affiliations:** Northern Arizona University, Flagstaff, Arizona, United States

## Abstract

The United States is experiencing growth in populations from culturally diverse backgrounds. Studies suggest that Latinx and American Indians experience chronic conditions such as cancer, heart disease, and diabetes in greater numbers than whites. Literature also suggests that Latinx and American Indian families play a significant role as informal caregivers for loved ones with chronic illness. However, little information is available about interventions to assist these patient-family caregiver dyads cope. The purpose of this systematic review is to synthesize published studies about psychosocial interventions developed or adapted for Latinx and American Indian care dyads in order to determine: (1) the benefits of these interventions; (2) their distinguishing features or adaptations, and; (3) recommendations for future intervention development. The protocol for this review was registered in advance with the International Prospective Register of Systematic Reviews (PROSPERO). We searched the databases CINAHL, PsycINFO, MEDLINE, and PubMeb using MeSH-derived keywords developed in consultation with a research librarian. Studies were included/excluded based upon pre-specified criteria. Three-hundred thirty-five records were identified, screened by the research team, and tracked according to PRISMA guidelines. After removing duplicates (n=9), studies that did not pertain to the conditions of interest (n=13), and studies that did not meet inclusion criteria (n=305), eight studies remained. Relevant information was abstracted from the final studies and synthesized by the research team. The majority of interventions for these populations are in cancer. Findings about benefits are largely inconclusive. Adaptations include a focus on cultural contexts, as well as culturally-based strengths, caregiving norms, and values.

